# Fish Oil for Healthy Aging: Potential Application to Master Athletes

**DOI:** 10.1007/s40279-021-01509-7

**Published:** 2021-09-13

**Authors:** Caoileann H. Murphy, Chris McGlory

**Affiliations:** 1 Department of Agrifood Business & Spatial Analysis, Teagasc Food Research Centre, Ashtown, Dublin 15, Ireland; 2 School of Kinesiology and Health Studies, Queen’s University, 28 Division St, Kingston, ON Canada

## Abstract

Master athletes perform high volumes of exercise training yet display lower levels of physical functioning and exercise performance when compared with younger athletes. Several reports in the clinical literature show that long chain *n-3* polyunsaturated fatty acid (LC *n*-3 PUFA) ingestion promotes skeletal muscle anabolism and strength in untrained older persons. There is also evidence that LC *n*-3 PUFA ingestion improves indices of muscle recovery following damaging exercise in younger persons. These findings suggest that LC *n*-3 PUFA intake could have an ergogenic effect in master athletes. However, the beneficial effect of LC *n*-3 PUFA intake on skeletal muscle in response to exercise training in both older and younger persons is inconsistent and, in some cases, generated from low-quality studies or those with a high risk of bias. Other factors such as the choice of placebo and health status of participants also confound interpretation of existing reports. As such, when considered on balance, the available evidence does not indicate that ingestion of LC *n*-3 PUFAs above current population recommendations (250–500 mg/day; 2 portions of oily fish per week) enhances exercise performance or recovery from exercise training in master athletes. Further work is now needed related to how the dose, duration, and co-ingestion of LC *n*-3 PUFAs with other nutrients such as amino acids impact the adaptive response to exercise training. This work should also consider how LC *n*-3 PUFA supplementation may differentially alter the lipid profile of cellular membranes of key regulatory sites such as the sarcolemma, mitochondria, and sarcoplasmic reticulum.

## Key Points

**Table:** 

LC *n*-3 PUFAs play important structural and biochemical roles in skeletal muscle phospholipid membranes.
Researchers have increasingly focused on the role of LC *n*-3 PUFAs in promoting skeletal muscle anabolism in older adults.
There is insufficient evidence to warrant LC *n*-3 PUFA intake above population recommendations to enhance the adaptive response of skeletal muscle to exercise training in older adults.
Future work should focus on the interplay between LC *n*-3 PUFAs and protein ingestion during exercise training as well as the incorporation of LC *n*-3 PUFAs into various cellular membranes.

## Introduction

The population of the world is expected to grow from just over 7 billion in 2019 to 9.7 billion by the year 2050 [1]. Simultaneously, the number of people over 65 years of age will more than double, from 703 million to 1.5 billion [1]. This shift towards an aging population poses some major challenges. Aging is associated with increased risk of cardiovascular disease [2], type 2 diabetes [3], and reduced physical functioning [4], the treatment of which imposes a large economic burden [5]. An often-overlooked consideration is that older persons also play numerous positive roles in society, including volunteering, childcare, as well as imparting important cultural and social influences [6, 7]. Thus, strategies that support ‘healthy aging’ by offsetting disease and disability in older persons will have important personal, economic, and societal consequences.

Since the early work in the 1940s of Captain Thomas De Lorme who employed progressive resistance exercise to restore muscle power in poliomyelitis patients [8] to contemporary resistance exercise protocols that enhance physical performance in the athletic setting [9], resistance exercise training has long been used as a means to improve human health and physical functioning. Similarly, endurance exercise training is often prescribed to improve metabolic and cardiovascular health, and indices of performance for a variety of sports (e.g., marathon running, soccer, cycling) [10]. Given the increased number of older persons worldwide together with the purported health promoting effects of exercise training, it is unsurprising that the number of older persons engaging in exercise training has risen [11]. Of this older subset of the population, there are a unique group who regularly engage in exercise training, known as ‘master athletes’. Master athletes are defined as active athletes aged 35 years and above, but typically refers to older athletes who train or compete for organized sporting events [12, 13]. Master athletes are widely considered an exemplar of healthy aging as they exhibit remarkable physical and physiological function relative to their sedentary counterparts [13, 14]. Some master athletes even display similar levels of muscular strength, mass and morphology, and aerobic capacity to that of untrained younger persons, highlighting the potency of exercise training in mitigating the adverse effects of aging [13, 14]. Yet, despite high levels of physical activity that partially protect against the decline in musculoskeletal and cardiorespiratory health with advancing age [13–17], master athletes still exhibit lower levels of exercise performance compared to their trained, younger counterparts [13, 17–19].

A key factor supporting the adaptive response to exercise training in both younger and master athletes is adequate nutrition. Most research in this area centers on the role of carbohydrates and protein [20–23]. However, in recent years, there has been increased focus on fatty acids; specifically, long-chain *n-3* polyunsaturated fatty acids (LC *n*-3 PUFAs) [24, 25]. Although traditionally associated with improved cardiovascular health [26], there is mounting, albeit equivocal evidence, that LC *n-3* PUFA-enriched fish oil ingestion influences recovery from acute and chronic exercise training in young persons [24, 25]. These data [24, 25] are complemented by a small number of clinical reports in older adults, suggesting that supplementation with LC *n*-3 PUFAs increases skeletal muscle mass and/or strength both in free-living environments [27] and in response to resistance exercise training [27, 28]. Given that, despite high training volumes, master athletes exhibit age-related decrements in exercise performance [13, 19] together with the knowledge that they may experience impaired adaptive responses [29, 30] and recovery [31] following exercise compared to younger adults, LC *n*-3 PUFA intake could have utility for promoting exercise adaptation in master athletes. In the present review, we draw upon data from both the athletic and clinical settings to critically evaluate the potential of LC *n*-3 PUFA intake to promote performance in, and recovery from, exercise training in master athletes. We also suggest areas for future experimental research and conclude by making practical recommendations for LC *n*-3 PUFA intake in older, exercising populations. The focus of our paper is primarily on older persons, so the interested reader is instead referred to excellent recent reviews on this topic in younger persons [24, 25].

## Impact of Aging on Skeletal Muscle Protein Turnover

Skeletal muscle mass and strength are inversely associated with mortality and play important roles in supporting athletic performance across a range of sporting events [32–34]. Skeletal muscle mass is regulated by the balance between rates of muscle protein synthesis (MPS) and muscle protein breakdown (MPB). Both MPS and MPB are highly sensitive to nutritional and contractile *stimuli* (i.e., dietary protein intake, resistance exercise) [35]. For instance, the ingestion of dietary protein leads to hyperaminoacidemia that transiently increases MPS rates and, predominantly via the accompanying hyperinsulinemia, suppresses MPB rates resulting in a positive net muscle protein balance. Resistance exercise sensitizes skeletal muscle to hyperaminoacidemia resulting in a potentiation of MPS and a protracted state of positive protein balance compared with protein ingestion alone [36]. Thus, repeated bouts of resistance exercise paired with protein ingestion results in skeletal muscle hypertrophy. Despite a strong relationship between skeletal muscle mass and strength [37], changes in muscle mass with resistance exercise training may not always predict changes in strength [38].

Unfortunately, older adults exhibit anabolic resistance whereby the MPS response to dietary protein intake and resistance exercise is blunted compared with younger adults [39, 40]. Anabolic resistance contributes to age-related muscle mass loss and a lower hypertrophic response to resistance training in older compared to younger adults [41–43]. Even highly active master athletes show lower integrated rates of MPS compared to younger athletes during intense endurance training paired with high protein intake [30]. They also exhibit comparable integrated rates of MPS to older untrained adults both at rest and following resistance exercise [29].Therefore, identifying nutritional strategies beyond protein ingestion that overcome anabolic resistance is key to supporting both health and exercise adaptations in both master athletes and the general older population alike.

## LC *n*-3 PUFAs and Skeletal Muscle Adaptation to Resistance Exercise Training

The most well-studied LC *n*-3 PUFAs are eicosapentaenoic acid (EPA) and docosahexaenoic acid (DHA), which are found in fish oils and many over-the-counter fish oil supplements. The incorporation of EPA and DHA into the phospholipid membrane of tissues, and the subsequent production of a variety of lipid and anti-inflammatory resolving mediators, is thought to promote beneficial effects in humans [44]. Although often linked with improved cardiovascular and neurological function, there is increasing evidence that EPA and DHA ingestion positively impact skeletal muscle [45, 46].

Smith and colleagues conducted the initial seminal human studies that examined the influence of LC *n*-3 PUFAs on human skeletal muscle [47–49]. In the first two trials, LC *n*-3 PUFA supplementation (1.9 g/day EPA + 1.5 g/day DHA for 8 weeks) increased EPA and DHA content in skeletal muscle phospholipids and augmented the acute (3 h) mixed MPS response to a hyperaminoacidemic–hyperinsulinemic clamp in untrained younger [48] and older men and women [49]. A subsequent study reported increased thigh muscle volume and strength after 6 months of the same dose of LC *n-3* PUFAs in sedentary older adults [47] with impressive treatment effects [∆ muscle volume: 3.6% (95% CI 0.2%, 7.0%); ∆ handgrip strength: 2.3 kg (95% CI 0.8, 3.7 kg)]. Others have also shown that supplementation with LC *n*-3 PUFAs potentiated strength gains [27] and muscle quality (unit strength as a function of unit area) [28] following resistance exercise training in older women. Collectively, these data [27, 28, 47–49] suggest that LC *n*-3 PUFA intake increases the EPA and DHA content of skeletal muscle in older adults leading to enhanced muscle anabolism and/or function.

While there are reports of enhanced rates of MPS, skeletal muscle mass, and strength with LC *n*-3 PUFA intake in older adults [27, 28, 47–49], not all studies have shown a positive effect. Indeed, in a recent study in 107 older adults not engaged in exercise training, consuming 1.6 g/day EPA + 2.3 g/day DHA for 6 months—provided as part of a mixed macronutrient drink containing leucine-enriched protein—failed to impact appendicular lean mass, handgrip strength or knee extensor strength [50]. Moreover, this intervention [50] did not improve physical performance in the form of the short performance physical battery (SPPB) and the timed up-and-go test. Similarly, another study involving over 2000 older adults [51] did not detect an influence of 3 years of LC *n*-3 PUFA supplementation (0.3 g EPA/day + 0.7 g DHA/day) alone, or when combined with home-based strength training (30 min × 2 days/week, no load progression), on the primary outcome of SPPB test score. The reasons for why these findings [51] differ from previous reports [27, 28, 47] are unclear, but a major concern is that many of the participants in the aforementioned study [51] had baseline SPPB scores near-maximal (median: 11 out of a maximum score of 12 [interquartile range 10–12]). As such there was clearly minimal scope for improvement which may have masked potential beneficial effects of the interventions. It is also important to note that the study of Rodacki et al. [27], in which LC *n*-3 PUFAs potentiated strength gains [27], lacked a placebo group, whereas in the study of Da Boit et al. [28], the positive effects of LC *n*-3 PUFA intake on muscle quality were only observed in women and not men, suggesting a sex-based effect of LC *n*-3 PUFA intake on skeletal muscle quality. Another possibility is that the participants in the studies reporting no advantageous effects of LC *n*-3 PUFA supplementation were consuming sufficient protein, precluding the ability to observe an ergogenic effect of LC *n*-3 PUFA intake. Although most of the LC *n*-3 PUFA supplementation studies do not report protein intake [28, 47, 51], protein intakes were higher (~ 1.3 g/kg/day) among participants in the study by Murphy et al. [50], which reported no favorable effects of LC *n*-3 PUFA, compared to ~ 0.9 g/kg/day in the study by Rodacki et al. [27]. Future work that examines how LC *n*-3 PUFA intake and resistance exercise training influence skeletal muscle mass, strength, and physical performance in older women and men may resolve these discrepancies and provide important information for the field. In this regard, we note that the larger trial of Bischoff-Ferrari et al. [51] examined multiple related secondary outcomes, including muscle mass, handgrip strength, and five times sit-to-stand performance, which will presumably be reported in future publications.

The mechanisms underlying reports of enhanced muscle anabolism with LC *n*-3 PUFA intake remain unknown. One working hypothesis is that supplementation with LC *n*-3 PUFAs increases the EPA and DHA content of skeletal muscle potentiating rates of mixed MPS in response to amino acid provision [48, 49] leading to increases in skeletal muscle mass and strength [27, 28, 47]. However, both Da Boit et al. [28] and Murphy et al. [50] observed no effect of LC *n*-3 PUFA supplementation on integrated myofibrillar MPS rates measured over 3–4 days. McGlory et al. [52] also failed to detect an influence of 8 weeks of 3.5 g/day EPA and 0.9 g/day DHA on acute (3 h) rates of myofibrillar MPS in response to oral protein feeding with or without resistance exercise in young men [48, 49]. There are several potential explanations for why these findings oppose the working hypothesis. First, it is perhaps unsurprising that Da Boit et al. [28] and Murphy et al. [50] did not detect changes in rates of myofibrillar MPS, as neither study observed a change in muscle mass, and the improvement in muscle quality observed by Da Boit et al. [28] was driven by an increase in muscle strength. Additionally, McGlory et al. [52] used a 30 g dose of whey protein, a dose known to saturate rates of myofibrillar MPS in the young population under investigation [53]. To our knowledge, only one study has shown that LC *n*-3 PUFAs increase rates of myofibrillar MPS in older persons following resistance exercise [54]. Yet that report [54] was limited to a 3 h capture in the fasted state 15 h following the cessation of exercise. The rates of myofibrillar MPS reported were also incongruent with what would be expected in this population, suggesting that additional, replication studies are required.

**Fig. 1:**
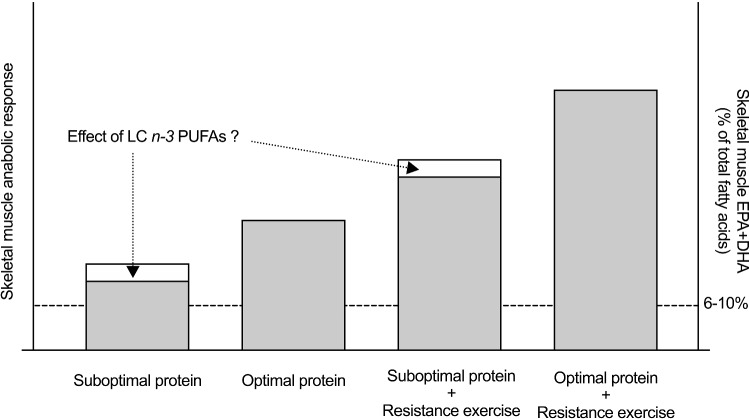
Theoretical construct of how long-chain *n-3* polyunsaturated fatty acids (LC *n*-3 PUFAs) influence skeletal muscle anabolism. Supplementation with LC *n*-3 PUFAs achieves a 6–10% eicosapentaenoic acid (EPA) + docosahexaenoic acid (DHA) enrichment of total fatty acids in skeletal muscle phospholipids. Enrichment of EPA + DHA in skeletal muscle phospholipids potentiates the anabolic response of skeletal muscle in response to sub-optimal, but not optimal, doses of protein ingestion at rest and following resistance exercise

In summary, several questions related to the interplay between protein dose and the anabolic response of skeletal muscle following LC *n*-3 PUFA intake remain unanswered. Specifically, can LC *n*-3 PUFAs potentiate acute rates of myofibrillar MPS and/or alter rates of MPB in response to a sub-optimal dose of oral protein ingestion? If so, does this potentiation lead to enhanced gains in skeletal muscle mass and strength with resistance exercise training (Fig. 1)? Given that older adults require a greater relative dose of protein intake to maximally stimulate rates of myofibrillar MPS compared to younger adults [39], studies that directly address these questions may influence the nutritional practices of master athletes.

## Recovery from Exercise-Induced Muscle Damage

Another proposed role for increased LC *n*-3 PUFA intake among athletes is attenuating the loss of muscle force and improving recovery following damaging resistance exercise. Many athletic events result in transient ultrastructural myofibrillar disruption, leading to impairments in muscular strength and power lasting for several days post-exercise—termed exercise-induced skeletal muscle damage (EIMD) [55]. EIMD is frequently accompanied by delayed onset of muscle soreness (DOMS), local muscular inflammation, swelling, and reduced range of motion of the affected limb [55]. Although there is some concern that interfering with recovery processes may blunt the adaptive response to exercise training [56], improved recovery from the side effects of damaging exercise could be beneficial for athletes in situations when adaptation is secondary to recovery and performance (e.g., tournaments). Interventions that expedite recovery following EIMD may be especially relevant among master athletes, who can experience slower muscle recovery compared to their younger counterparts [31].

Systematic reviews report generally repeatable, positive effects of LC *n*-3 PUFA supplementation on markers of muscle recovery (e.g., muscle soreness, range of motion, and swelling) and strength preservation in younger athletes [24] and younger, healthy individuals [25]. These effects are proposed to be due to the incorporation of EPA and DHA into membrane phospholipids leading to the production of anti-inflammatory and pro-resolving mediators as well as analgesic effects. However, despite the potential for LC *n*-3 PUFA supplementation to attenuate indices of muscle damage in younger persons, to our knowledge, no study has examined whether LC *n*-3 PUFA intake alters indices of muscle damage in older persons. It is also important to highlight that a recent study questioned the scientific quality of many of the previous trials examining the impact of LC *n*-3 PUFA supplementation on recovery following EIMD in younger people [57]. Indeed, this systematic review has highlighted an unsatisfactory risk of bias among many studies due to flaws in the design, conduct, analysis, and reporting of outcomes with further key criticisms including failure to account for the baseline LC *n*-3 PUFA status, changes in tissue LC *n*-3 PUFA content, and inappropriate intervention duration [57]. As there is considerable variation between existing studies in LC *n*-3 PUFA supplement dose, composition (i.e., relative content of EPA and DHA) and duration between studies, it is currently not possible to make recommendations for applied practice [57]. Thus, future work in older adults that addresses these important experimental considerations would add to existing data in younger adults and assist in the development of nutritional interventions to attenuate and promote recovery from EIMD in master athletes [31].

## LC *n-3* PUFA and Endurance Exercise Training

Although a significant body of research has focused on the potential for LC *n*-3 PUFA intake to alter the adaptive response of skeletal muscle to resistance exercise, comparably fewer studies have focused on the potential of LC *n*-3 PUFA to impact adaptations to endurance exercise. Interestingly, evidence for an ergogenic effect of LC *n*-3 PUFAs on endurance exercise performance can be found in the animal kingdom. The semipalmated sandpiper (*Calidris pusilla*) gorges on small burrowing amphipods that are enriched with LC *n*-3 PUFAs in the Bay of Fundy, Canada, prior to embarking on the long migratory journey south (4500 km in 3 days) to the northern coast of South America [58]. This form of natural doping with LC *n*-3 PUFAs enhances the expression of mitochondrial genes supporting lipid metabolism leading to optimal flight performance. In humans, supplementation with EPA + DHA in young men alters mitochondrial bioenergetic function [59, 60] that is linked to reduced heart rate and oxygen cost of cycling exercise [61, 62]. Ingestion of LC *n*-3 PUFAs also increased mitochondrial gene expression in older adults [63]. Yet, these underlying cellular effects of LC *n*-3 PUFAs rarely translate to improved endurance performance in humans [24]. As such, there is currently little rationale to recommend that individuals, older or younger, employ LC *n*-3 PUFA supplementation as an ergogenic aid to enhance endurance exercise performance. If LC *n*-3 PUFA intake proves to be efficacious in enhancing recovery from EIMD in older adults, it is plausible that supplementation with LC *n*-3 PUFA could improve recovery from activities such as downhill trail running [31]. Clearly, experiments are needed to test this hypothesis in master athletes engaging in endurance exercise.

## Impact of Placebo Choice

As with nearly all nutritional supplementation studies, choosing an appropriate placebo is a critical component of study design. In an ideal scenario, the placebo should be energy-matched but have no bearing on study outcomes, so that any changes in outcomes can be attributed to the intervention treatment. However, studies examining the influence of LC *n-3* PUFAs on skeletal muscle often employ a variety of placebos such as corn oil [47–49], coconut oil [52], sunflower oil [60, 64], or a mixture of oils [50] all of which contain unique, biologically active fatty acids (e.g., saturated fatty acids, monounsaturated fatty acids, and long-chain *n-6* fatty acids). In sufficient doses, these fatty acids impart differential effects on a multitude of biological processes such as the production of pro vs*.* anti-inflammatory signaling molecules as well as changes in skeletal muscle phospholipid composition [45]. Thus, it is possible that the selection of a given placebo could impact the outcome of a study particularly when the placebo contains fatty acids known to have opposite effects to the intervention. An alternative option would be to employ an inert placebo such as mineral oil that is an odorless mixture of alkanes and has been extensively used in the cardiovascular and pre-clinical literature. Despite concerns that high-dose mineral oil ingestion (4 g/day) is associated with increased concentration of inflammation markers (e.g., high-sensitivity C-reactive protein [hsCRP]) as well as low density lipoprotein (LDL) [26], a recent review of 80 studies indicated that mineral oil ingestion had either inconsistent or no clinical impact on either hsCRP or LDL [65]. Furthermore, aside from inducing a mild laxative effect, mineral oil results in minimal adverse events [65]. By employing inert compounds as placebos, future work may obviate issues linked with biologically active placebos. Although access to high-quality, pharmaceutical grade inert oils is not always possible, the choice of placebo should be an important consideration when designing and critically evaluating research in this field.

## Optimal Dose and Incorporation of LC *n-3* PUFA

Population recommendations for EPA and DHA intake vary from country to country but are typically 250–500 mg/day for EPA and DHA combined [66–68], with intakes of up to 5 g/day generally considered safe for human consumption [69]. Studies examining the potential ergogenic benefit of EPA + DHA intake typically use substantially higher doses than the population recommendations. Indeed, investigations reporting beneficial effects of LC *n*-3 PUFA supplementation on resistance training adaptations in older adults have used doses in the range of 0.7–2.7 g EPA + DHA [27, 28]. Even higher doses have been used in studies reporting improvements in rates of MPS, skeletal muscle mass, and strength without concomitant exercise (3.4 g EPA + DHA) [47, 49]. Nonetheless, the optimal LC *n*-3 PUFA dose required to elicit putative benefits related to exercise performance is unknown. What is established is that the beneficial influence of EPA and DHA intake on skeletal muscle is linked to changes in the LC *n*-3 PUFA skeletal muscle phospholipid profile. Using standard gas–liquid chromatography techniques, it requires at least 2 weeks of supplementation with 3.5 g/day EPA and 0.9 g/day DHA to detect a change in the EPA + DHA content of whole skeletal muscle in young men [70]. No study has established a time-course of the change in skeletal muscle lipid profiles with LC *n*-3 PUFA intake in older adults. Moreover, no study has established if a dose–response exists between LC *n*-3 PUFA intake and changes in the LC *n*-3 PUFA composition of skeletal muscle. Whether such a time-course of change and/or dose response would differ in older compared to younger adults is not known, but such a comparison would help to inform LC *n*-3 PUFA supplementation protocols in exercising older and younger persons.

While time-course data indicate that at least 2 weeks of ~ 4 g/day LC *n*-3 PUFA ingestion is required to increase the EPA + DHA composition of skeletal muscle [70], it should be acknowledged that these data are indicative of whole muscle and not fraction-specific phospholipids (i.e., sarcolemmal vs. mitochondrial). This point is important, because it is known that the EPA and DHA composition of mitochondrial and sarcolemmal phospholipids exhibits unique responses to LC *n*-3 PUFA intake in young adults [71], although the impact on specific phospholipid membranes has not been tested in older adults. The knowledge that the mitochondrial and sarcolemmal phospholipids are differentially affected by LC *n*-3 PUFAs opens the intriguing possibility that other important cellular phospholipid membranes may also display distinct responses to LC *n*-3 PUFAs. One interesting area worthy of future work is the impact of LC *n*-3 PUFA ingestion on the sarcoplasmic reticulum and Ca^2+^ handling, which is a vitally important aspect of muscle contraction and may be negatively impacted by aging [72]. While initial evidence suggests that 12 weeks of 5 g/day LC *n*-3 PUFA intake in healthy older men does not influence aspects of sarcoplasmic reticulum function [73], changes in sarcoplasmic reticulum or skeletal muscle phospholipid membrane lipid composition in that study were not directly assessed. However, the increase in blood EPA profiles is generally consistent with what would be expected based on the previous reports in humans in which skeletal muscle phospholipid EPA composition was increased [28, 49]. In contrast with a large body of work in animals, there are limited data regarding the influence of LC *n*-3 PUFA intake on sarcoplasmic reticulum lipid composition and function in humans and this warrants further study.

## Sources of LC *n-3* PUFAs

Although the ergogenic impact of LC *n*-3 PUFAs on the adaptive response to exercise training is equivocal, it is clear that consumption of adequate LC *n*-3 PUFAs is important for health [66–68]. Furthermore, foods rich in LC *n*-3 PUFAs such as fish contain other key nutrients such as essential amino acids and micronutrients that are often lacking in the diet of older adults (Table 1). Most fish contain EPA and DHA; however, the content varies considerably between species and is highest in oily fish such as salmon and mackerel, intermediate in shellfish such as oysters and mussels, and lower in white fish such as cod and haddock. Fresh tuna, which was previous categorized as an oily fish, was recently reclassified as a non-oily fish in the UK following new data showing that its LC *n*-3 PUFA concentrations are comparable to that found in most white fish [74]. The EPA and DHA content also varies within a species depending on environmental variables and whether it is wild or farmed [75]. Provided that farmed fish are raised using appropriate feeds, their LC *n*-3 PUFA content is comparable to, or even higher than, wild fish [75]. Although a growing number of functional foods are being enriched with LC *n*-3 PUFAs (e.g., eggs, peanut butter, orange juice, margarine, bread, yogurt, and milk), fish still represents the primary dietary source [76]. As an alternative to whole foods, LC *n*-3 PUFA can be consumed via supplements. LC *n*-3 PUFA supplements vary in their EPA + DHA content ranging from ~ 150 to 1300 mg per capsule [77, 78]. Thus, it would be wise to examine the EPA and DHA content of supplements marketed as being enriched with LC *n*-3 PUFA prior to purchase.

**Table 1:** Content of LC *n*-3 PUFA and selected nutrients in fish^a^

	Per 100 g							
Food	EPA (mg)	DHA (mg)	Energy (kcal)	Protein (g)	Vitamin D (% RDA)^b^	Vitamin B12 (% RDA)	Selenium (% RDA)	Household measure
Mackerel, baked	968	1568	239	24	115	325	95	1 small fillet = 170 g
Herring, smoked, kippered	970	1179	217	25	15	779	96	1 small fillet = 20 g
Anchovies, canned in oil, drained	763	1292	210	29	11	37	124	1 anchovy = 4 g
Salmon, canned in water, drained	370	687	137	21	100	193	62	1 regular can, drained = 177 g
Sardines, canned in oil, drained	473	509	208	25	32	371	96	1 regular can, drained = 92 g
Squid, steamed	291	681	183	31	0	97	162	1 cup = 140 g
Trout, steamed	273	649	117	25	133	192	54	1 small fillet = 113 g
Mussels, steamed	374	504	171	24	0	896	162	1 medium mussel = 8 g
Salmon, baked	229	419	160	26	91	196	72	1 small fillet = 170 g
Oysters, raw	177	136	51	6	0	365	36	1 eastern oyster = 14 g
Halibut, baked	83	161	115	23	39	52	104	1 small fillet = 170 g
Crab, baked	124	82	102	22	0	162	96	1 cup = 118 g
Lobster, steamed	116	77	88	19	0	35	132	1 medium lobster, no shell = 295 g
Prawns, steamed	87	89	91	17	1	35	69	1 cup = 145 g
Haddock, steamed	53	112	93	21	4	82	59	1 small fillet = 170 g
Cod, baked	43	121	87	19	4	93	52	1 small fillet = 170 g
Tuna, canned in oil, drained	27	101	198	29	45	92	138	1 regular can, drained = 160 g
Scallops, baked	52	75	85	15	0	68	29	1 scallop = 13 g
Tuna, fresh, steamed	15	111	137	31	14	93	207	1 small fillet = 170 g
Catfish, steamed	21	72	150	19	2	129	19	1 small fillet = 113 g

*DHA* docosahexaenoic acid, *EPA* eicosapentaenoic acid, *RDA* recommended daily allowance

^a^Values derived from the Food and Nutrient Database for Dietary Studies 2017–2018 on FoodData Central [88]. Data are sorted based on content of combined EPA + DHA per 100 g. % RDA values are based on the RDA for adults aged 51–70 years; vitamin D 15 µg/day [89], vitamin B12 2.4 µg/day [90], selenium 55 µg/day [91]

^b^For adults aged 71 years and older the RDA is higher (20 µg) [89]

## Potential Adverse Effects of Increased LC *n*-3 PUFA Intake

Notwithstanding the potential benefits of oily fish intake, investigators have raised concerns about contamination with mercury, dioxins, and polychlorinated biphenyls (PCBs) [67, 79, 80]. Levels of contaminants in seafood depend on several factors, including nature of the contaminant, fish species, harvest location, age, and composition of feed [79]. Numerous governmental bodies have acknowledged that the benefits of fish consumption outweigh risks of contamination at the recommended intake (i.e., 2 portions per week) [67, 79]. Obtaining the high LC *n*-3 PUFA dose used in several studies reporting strength and muscle mass benefits in older adults (2.7–3.4 g/day) [28, 47] would necessitate the intake of ~ 8 to 20 portions of oily fish per week. While contamination risks are lower in older adults compared to at-risk groups (i.e., children, women who are or may become pregnant), at such high intake levels contamination may be a concern, particularly if intake is largely derived from a single fish species [67]. Fish oil supplements generally contain little methylmercury [81]; however, some brands can be a source of PCBs and dioxins [82]. Furthermore, some [77, 83], but not all [78], studies have reported that a substantial proportion of LC *n*-3 PUFA supplements contain oxidized products exceeding industry-standard levels. Importantly, the impact of the oxidation of LC *n*-3 PUFA on biological functions and health consequences remains to be established [84, 85]. Another often anecdotally cited adverse effect of elevated LC *n*-3 PUFA intake is increased risk of bleeding. However, major randomized trials have demonstrated that even large doses of LC *n*-3 PUFAs (10 g/day EPA + DHA) do not affect bleeding risk [86] with one study showing that higher LC *n*-3 PUFA intake was associated with a reduced risk of bleeding [87].

## Future Directions and Conclusion

**Fig. 2:**
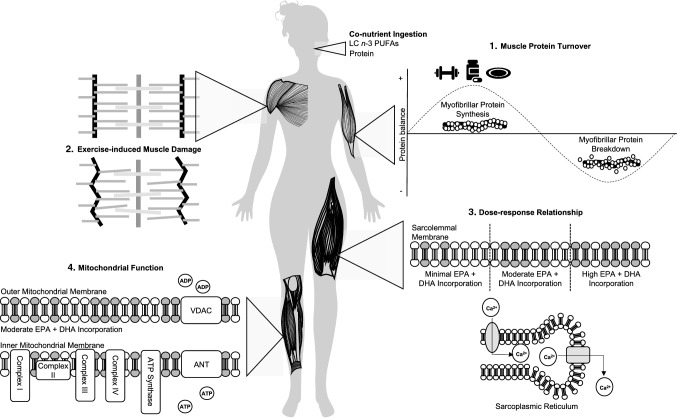
Schematic illustration of potential areas for further research in master athletes. **1.** Can co-ingestion of protein and long-chain *n-3* polyunsaturated fatty acids (LC *n*-3 PUFAs) influence muscle protein synthesis and muscle protein breakdown rates? **2.** What is the impact of LC *n*-3 PUFAs on skeletal muscle damage? **3.** Is there a dose–response relationship between LC *n*-3 PUFA intake and skeletal muscle? **4.** Can changes in mitochondrial protein expression/bioenergetic function with LC *n*-3 PUFA intake improve endurance exercise performance? *ADP* adenosine diphosphate, *ATP* adenosine triphosphate, *ANT* adenosine nucleotide translocator *Ca*^*2*+^ calcium, *EPA* eicosapentaenoic acid, *DHA* docosahexaenoic acid, *VDAC* voltage-dependent anion channel

Although much work has been done examining the potential for LC *n*-3 PUFAs to enhance the adaptive response to exercise training, there remain a number of unanswered questions (Fig. 2). First, despite reports of improved skeletal muscle mass and strength with LC *n*-3 PUFA intake [27, 28, 47] in older persons, there are a number of studies demonstrating no effect [50–52]. Reasons for these discrepancies could be differences in protein intake, baseline LC *n*-3 PUFA status of the participants, as well as differences in LC *n*-3 PUFA dosing between studies: all of which require further experimental attention. Another important goal for future research will be to establish whether a dose–response effect exists between LC *n*-3 PUFAs intake and changes in skeletal muscle LC *n*-3 PUFA composition and if these changes are modified by age. Such studies would clearly be relevant when designing supplementation strategies with a view to alter skeletal muscle LC *n*-3 PUFA profiles in older adults.

In conclusion, there is little evidence to suggest that ingestion of LC *n*-3 PUFAs above current population recommendations of 250–500 mg EPA + DHA/day enhances exercise performance in older persons. Although numerous studies report positive effects of LC *n*-3 PUFA supplementation on markers of muscle recovery and force preservation in younger adults, the scientific quality of many of those studies is sub-optimal and there are no data on older individuals. Future work that addresses the influence of habitual protein intake, baseline LC *n*-3 PUFA status, and the LC *n*-3 PUFA dose on the response to LC *n*-3 PUFA supplementation would add to existing literature and may inform future nutritional guidelines aimed at promoting the adaptive response of skeletal muscle to exercise training in master athletes.
